# Race- associated molecular differences in uterine serous carcinoma

**DOI:** 10.3389/fonc.2024.1445128

**Published:** 2024-10-03

**Authors:** Olivia D. Lara, Hannah Karpel, Steven Friedman, Kari E. Hacker, Bhavana Pothuri

**Affiliations:** ^1^ Division of Gynecologic Oncology, Lineberger Comprehensive Cancer Center, University of North Carolina at Chapel Hill, Chapel Hill, NC, United States; ^2^ Department of Obstetrics and Gynecology, Division of Gynecologic Oncology, Laura and Isaac Perlmutter Cancer Center, New York University (NYU) Langone Health, New York, NY, United States; ^3^ Department of Population Health, New York University Langone Health, New York, NY, United States

**Keywords:** uterine cancer, health disparities, race, gynecologic cancer, targeted treatment

## Abstract

**Purpose:**

Endometrial cancer (EMCA) is the most common gynecologic malignancy, and new diagnoses are increasing in the United States. Black patients are more likely to present with advanced stage, be diagnosed with high-risk uterine serous carcinoma (USC) and die of their cancer.

**Methods:**

Patients with endometrial adenocarcinoma who received tumor FoundationOne CDx testing at our institution between January 2017 and August 2022 were identified. Genomic alterations, demographic and clinical characteristics were collected. Descriptive statistics and Fisher’s exact test were used to analyze data.

**Results:**

A total of 289 patients (29.4% Black and 52.6% White) with advanced or recurrent endometrial adenocarcinoma underwent FoundationOne CDx testing. USC comprised 26.3% (76 of 289) of tested tumors. Of USC tumors, 33 of 76 (44%) were of Black race. USC occurred more frequently in Black patients (33 of 85 [38.8%] Black patients compared to 30 of 152 [19.7%] White patients, p<0.05). Among USC, *CCNE1* amplification occurred more frequently in Black patients than in White patients (12 of 33 [36.36%] vs 2 of 30 [6.67%], p<0.05) while PI3K/AKT/mTOR pathway mutations occurred less frequently (16 of 33 [48.5%] vs 26 of 33 [86.7%], p=0.17). Among patients with *CCNE1* amplification 73.3% (11 of 15) progressed on or within 12 months of first-line platinum-based therapy. *CCNE1* amplification had significantly shorter median overall survival (97.3 months vs 44.3; HR (95%CI): 7.1 (10.03, 59.4) p< 0.05).

**Conclusions:**

Black patients constituted 44% of patients with USC in our study and had an increased frequency of *CCNE1* amplification. Patients whose tumors harbored *CCNE1* amplification had shorter overall survival. Identifying actionable mutations in this high unmet need population is crucial to improving outcomes among Black patients with uterine malignancy. Development of new targeted-therapies will need to keep these alterations at the forefront as trials are being designed.

## Introduction

Racial disparities in endometrial cancer outcomes are well documented (1, 2). When compared with White patients, Black patients are more likely to present at advanced stage and with nonendometrioid, high-risk histology. Only half of Black patients present with early stage disease whereas 73% of White patients present with stage I disease (3, 4). In the United States, Black women are twice as likely to die from endometrial cancer; age-adjusted mortality rates are higher among Black patients than among non-Hispanic White women (8.85 v 5.70 deaths per 100,000 women from 2000 to 2011). The percent increase in mortality is steeper among Black patients as well (annual percent change, 2009-2006 = 2.3 in Black patients vs 0.1 in White patients) (5, 6). These survival differences signify one of the most alarming examples of racial disparity in gynecologic oncology.

The etiology of health care disparities is multifactorial and can be attributed to social and biologic determinants of health. Social determinants of health include socioeconomic and cultural constructs, access to equitable healthcare, education and safe environmental living conditions. The differences in endometrial cancer mortality between Black and White patients vary substantially based on these factors. Black patients are less likely to receive guideline-concordant therapy, and have health care coverage or access to effective treatments (5). Among Black patients diagnosed with uterine cancer, neighborhood income, and insurance status were found to account for 7.2% and 11.5% of excess relative risk among Black patients less than 65 years of age (7). Despite adjusting for treatment, sociodemographic and histopathologic variables mortality among Black endometrial cancer patients remains higher (8). Furthermore, our understanding of tumor biology among Black patients is limited by our ability to enroll Black patients into clinical trials and offer tumor next-generation sequencing (9). Suboptimal race reporting in hallmark uterine cancer studies additionally contributes to our lack of understanding of tumor biology (10).

Through the use of tumor next-generation sequencing our understanding of tumor biology has grown substantially. Molecular profiling of tumors has led to the identification of targetable alterations among patients with endometrial cancer. Poor enrollment of Black patients in clinical trials and limited access to molecular tumor profiling among Black patients limits the discovery of actionable targets and drug development among minority patients. To address this gap we examined genomic profiles among Black patients with high-risk uterine serous carcinoma.

## Materials and methods

### Study design and patient population

We performed a retrospective cohort study including patients who underwent tumor next-generation tumor sequencing at our institution from January 2017 to August 2022. This study was approved by the institutional review board at New York University Langone Hospital. Tumors at our institution are submitted to Foundation Medicine Inc for next-generation sequencing, data analysis and annotation (11). Histologic subtype was determined by immunohistochemical staining, expert pathologist review and final results of next-generation sequencing. The final reports, including detected genomic findings, were reviewed retrospectively. Commonly altered genes, identified as those genes with greater than 15% alteration rate in our cohort, were included in our analysis.

Demographic and clinical information was obtained retrospectively using the electronic medical record. Collected data included age, race, ethnicity, insurance status, stage at diagnosis, recurrence history, lines of therapy and disease status. Race and ethnicity were self-reported. Our data included patients who identified as Black, White, Asian or Other race. A sub-analysis between Hispanic White patients and non-Hispanic White patients was not performed given small numbers and limited power. Progression free survival was defined from start of therapy to clinical progression or radiographic evidence of disease progression following completion of primary therapy. Overall survival was calculated using time from diagnosis to death or last follow-up.

### Statistical analysis

Descriptive statistics were utilized to describe the patient characteristics in the study cohort. Associations between genomic alteration frequency and clinical characteristics were tested using Fischer exact test to determine the association between two binary variables (White and Black race). Fischer’s exact test is used to determine if the proportions of categories in two group variables significantly differ from one another. Kaplan-Meier survival plots were interpreted using nonparametric Mann-Whitney test. Statistical significance was defined as p < 0.05. All statistical analyses were performed using SPSS statistics software.

## Results

### Patient cohort

**Figure 1:**
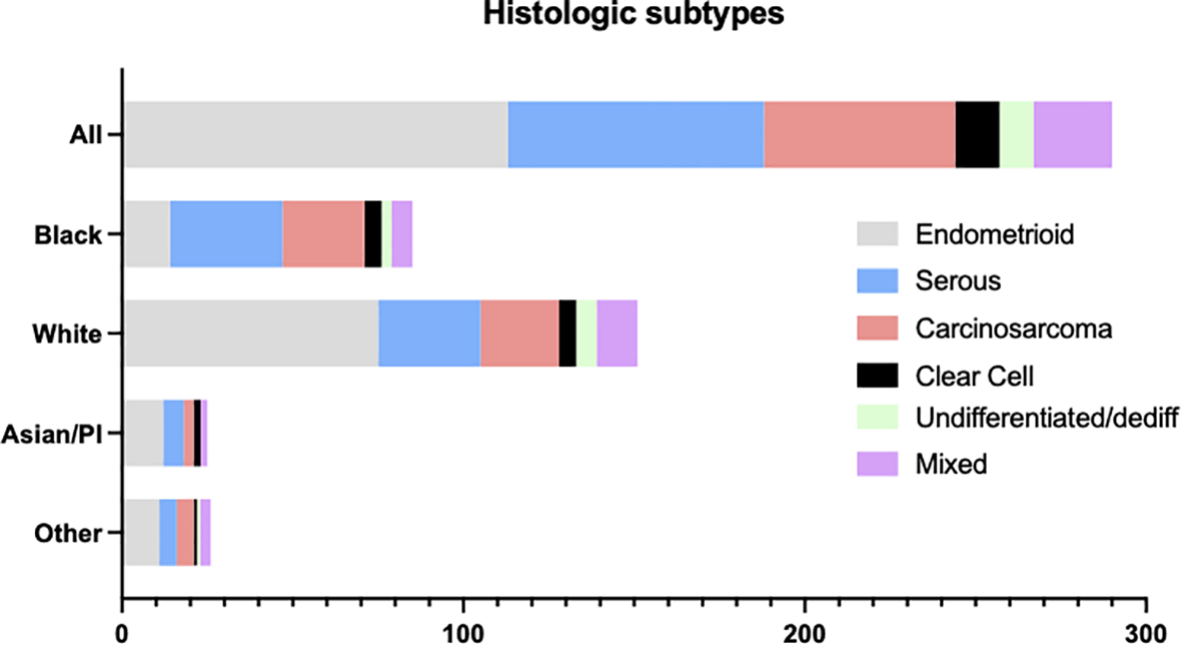
Endometrial carcinoma histologic subtypes.

A total of 289 patients with advanced or recurrent endometrial adenocarcinoma were referred for tumor next-generation sequencing (**Figure 1**). Of these, 76 (26.3%) were uterine serous carcinomas. The remaining tumors included 113 (39.1%) endometrioid and 56 (19.4%) carcinosarcoma. Mixed, clear cell, undifferentiated and dedifferentiated tumors comprised <10% of the cohort individually. We next examined histologic subtypes stratified by race (**Figure 1**). Among all endometrial adenocarcinomas, tumors from patients of Black race comprised 85 of 289 (29.4%) of tumors compared to tumors from patients of White race who compromised 152 of 289 (52.6%) of the cohort. Among histologic groups, tumors from patients of White race comprised most patients with endometrioid histology (75 of 113, [66.4%]). A greater percentage of tumors from patients of Black race were noted to have uterine serous carcinoma (33 of 85 [38.8%] Black patients compared to 30 of 152 [19.7%] White patients, p<0.05) and uterine carcinosarcoma (24 of 85 [28.2%] Black compared to 23 of 152 [15.1%] White, p=.07)

The baseline demographics of patients with uterine serous carcinoma were examined. The median age of cancer diagnosis in this population is 69 years (**Table 1**). In the overall cohort the majority of patients with advanced stage disease. This is due to the approval of tumor next generation sequencing for advanced or recurrent disease whereby early-stage patients are underrepresented in our cohort. No racial differences were seen among therapies including surgery and adjuvant radiation or chemotherapy. 12 of 33 (36.4%) Black patients died due to cancer-related causes compared to 33.3% (10 of 30) White patients (p=0.49).

**Table 1:** Baseline demographics in patients with Uterine Serous Carcinoma.

Characteristic	All n=75	White n=30	Black n=33	Asian/PI n=6	Other n=5	P-value*
**Age, median, y**	69 [64, 76]	72 [64, 79]	67.5 [62, 73]	67.5 [64, 74]	69 [64, 76]	0.12
Stage						0.28
** I/II, n (%)**	18 (23.7)	9 (30.0)	6 (18.2)	2 (33.3)	1 (16.7)	
** III/IV, n (%)**	57 (75.0)	21 (70.0)	27 (81.8)	4 (66.7)	5 (83.3)	
Surgery						1.0
** Yes, n (%)**	72 (94.7)	30 (100.0)	31 (93.9)	6 (100.0)	5 (83.3)	
** No, n (%)**	3 (3.9)	0 (0.0)	2 (6.1)	0 (0.0)	1 (16.7)	
Carbo/taxol						1.0
** Yes, n (%)**	71 (93.4)	29 (96.7)	32 (97.0)	5 (83.3)	5 (83.3)	
** No, n (%)**	4 (5.3)	1 (3.3)	1 (3.0)	1 (16.7)	1 (16.7)	
Radiation**						0.48
** Yes, n (%)**	32 (42.1)	19 (63.3)	8 (24.2)	1 (16.7)	4 (66.7)	
** EBRT n (%)**	19 (25.0)	9 (30.0)	6 (18.2)	0 (0.0)	4 (66.7)	
** Brachy n (%)**	16 (21.1)	12 (40.0)	2 (6.1)	1 (16.7)	1 (16.7)	
** No, n (%)**	43 (56.6)	11 (36.7)	25 (75.8)	5 (83.3)	2 (33.3)	
Status at last follow up						0.49
** Alive, n (%)**	47 (61.8)	20 (66.7)	20 (60.6)	2 (33.3)	5 (83.3)	
** Dead, n (%)**	24 (31.6)	10 (33.3)	12 (36.4)	2 (33.3)	0 (0.0)	
** Unknown, n (%)**	4 (5.3)	0 (0.0)	1 (3.0)	2 (33.3)	1 (16.7)	

*p-value Black vs White. **External beam radiation therapy (EBRT); vaginal brachytherapy (Brachy).

### Uterine serous carcinoma molecular alterations

**Figure 2:**
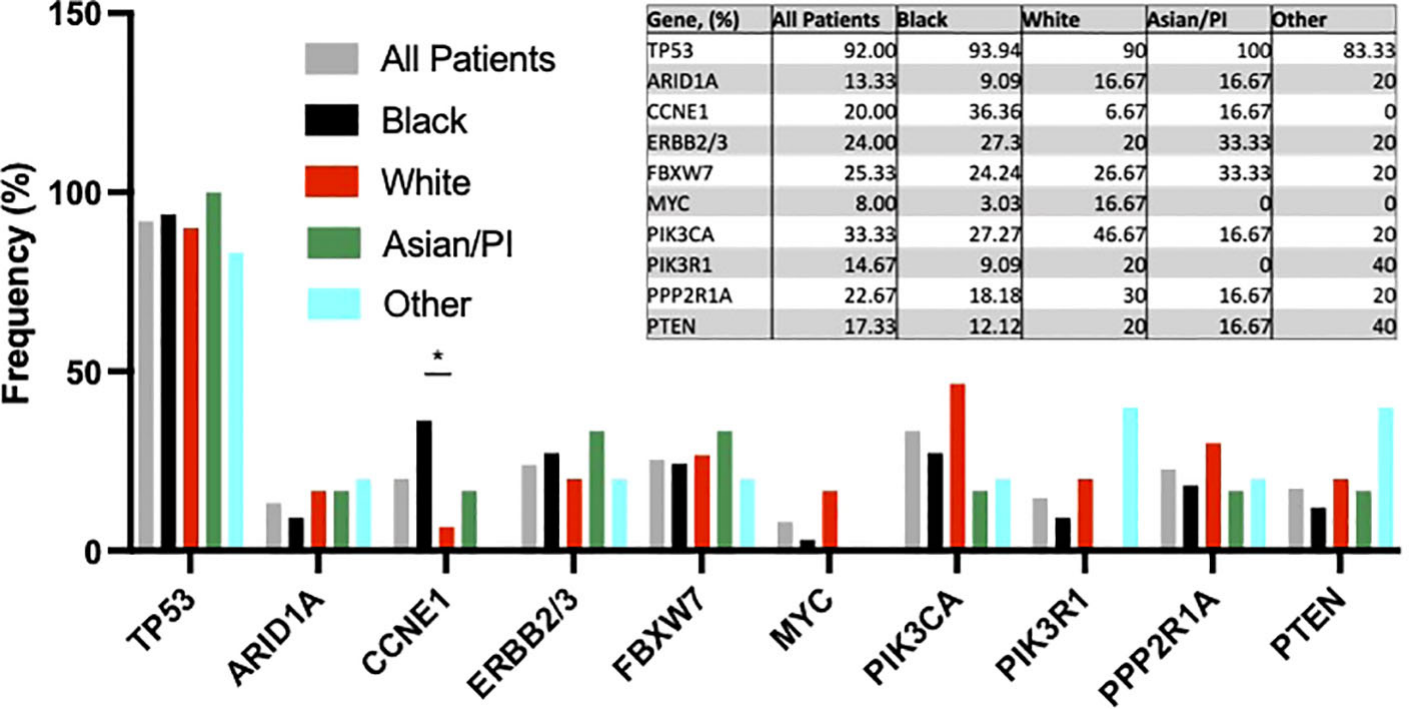
Genomic findings among uterine serous carcinoma tumors. *p-value <0.05.

Next, we examined tumor next generation sequencing results among women with uterine serous carcinoma. No racial differences in frequency of *TP53, ARID1A, ERBB2/3, FBXW7, MYC, or PPP2R1A* alterations were observed (**Figure 2**). PI3K/AKT/mTOR pathway mutations occurred less frequently (16 of 33 [48.5%] vs 26 of 33 [86.7%], p=0.17) in Black vs White patients. *CCNE1* amplification occurred more often in Black patients than in White patients (36.36% [12 of 33] vs 6.67% [2 of 30], p<0.05).

**Figure 3:**
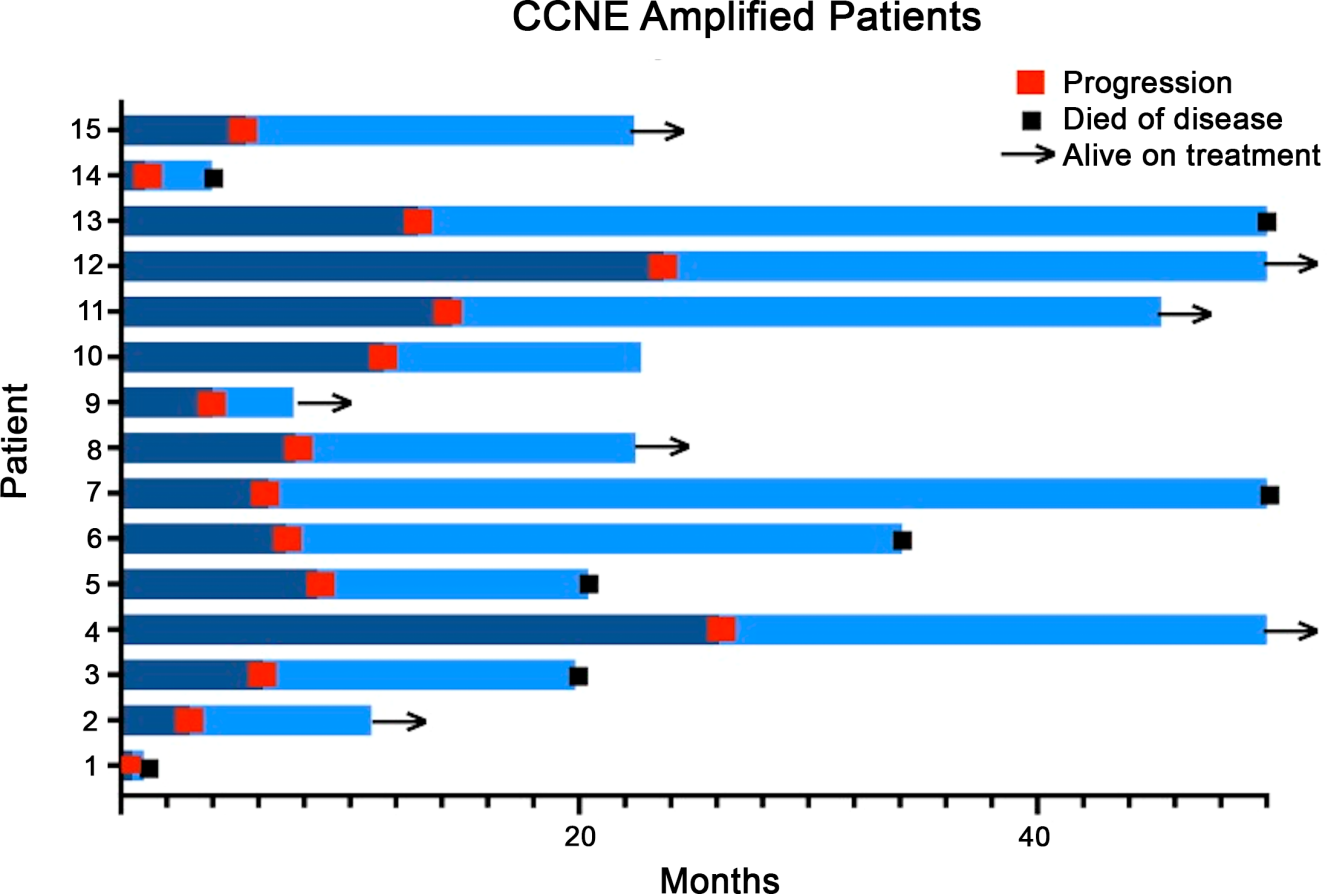
Outcomes of uterine serous carcinoma patients with *CCNE1* amplification.

**Figure 4:**
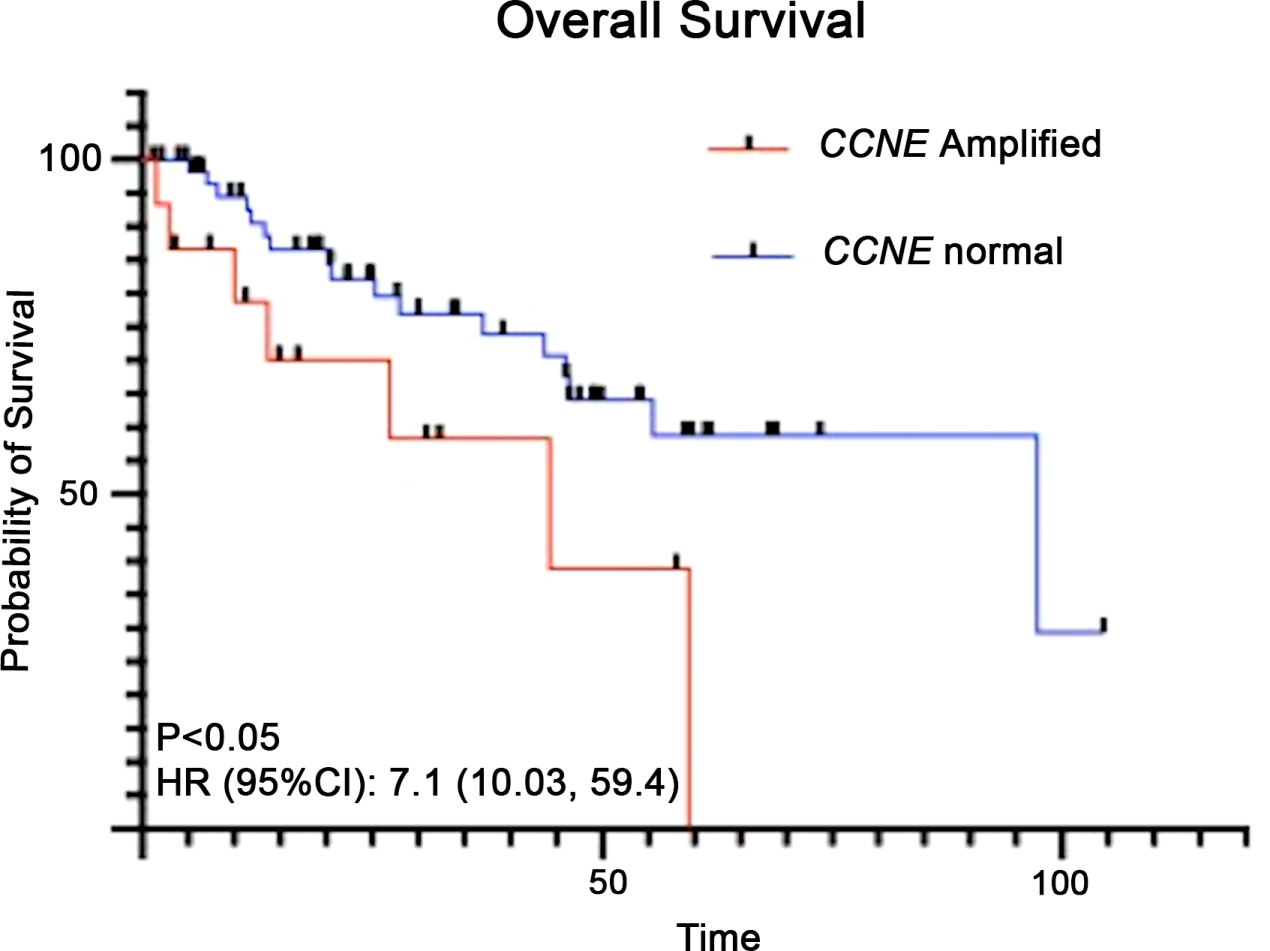
Kaplan-Meier analysis for overall survival of patients with uterine serous carcinoma and *CCNE1* amplification compared to those with normal *CCNE1* expression.

Clinical outcomes of patients with *CCNE1* amplifications were analyzed. Six of fifteen patients (40%) progressed while receiving first line platinum-based chemotherapy (**Figure 3**). Eleven of fifteen patients (73%) progressed within one year of diagnosis. In the survival analysis of all uterine serous carcinoma patients, those with *CCNE1* amplification had significantly shorter median overall survival (97.3 months vs 44.3; HR (95%CI): 7.1 (10.03, 59.4) p< 0.05) (**Figure 4**).

## Discussion

Endometrial cancer incidence is increasing and is projected to surpass ovarian cancer as the deadliest gynecologic cancer in the United States (12, 13). By 2040, endometrial cancer is projected to surpass colon cancer as the third leading cancer and fourth leading cause of cancer related death among women (14). Rates of aggressive nonendometrioid subtypes have increased among all women with a more pronounced increase in non-Hispanic Black women (15). In this study of advanced and recurrent endometrial carcinomas, we identified that Black patients were more likely than White patients to be diagnosed with high-risk endometrial subtypes (uterine serous carcinoma and uterine carcinosarcoma). We also discovered a higher rate of *CCNE1* amplification among Black patients compared to White patients with uterine serous carcinoma. *CCNE1* amplification was associated with shorter overall survival.

The majority of factors contributing to racial disparity in endometrial cancer are modifiable. This includes disparities in access to care, socioeconomic factors and inequities in treatment. The role of socioeconomic factors is difficult to quantify. Retrospective studies have found after adjusting for education, age, and tumor characteristics that patients without insurance had higher mortality risk compared to those patients with private insurance (16). Additionally after adjusting for socioeconomic and clinical factors the mortality risk among Black patients decreased from 2.35 (95%CI 2.20-2.51) to 1.28 (95% CI 1.17-1.40) (16). A Surveillance, Epidemiology, and End Results (SEER) database analysis among endometrial cancer patients found Black patients were 41% more likely than White patients to present with advanced-stage disease after controlling for age, tumor grade and histology (17). When stratified by aggressive versus nonaggressive endometrial tumors, among patients who had aggressive endometrial tumors, neither race or socioeconomic factors was associated with stage at diagnosis, suggesting socioeconomic factors may only impact outcomes in nonaggressive endometrioid tumors (17).

Aggressive endometrioid subtype, uterine serous carcinoma, accounts for 5% of endometrial cancers, but 40% of all endometrial cancer associated deaths (18). Analysis of Surveillance, Epidemiology and End Results (SEER) data including women with treated uterine corpus cancer identified a higher percentage of aggressive histologic variants including serous carcinoma among Black patients compared to White patients (12% vs 5%, p<0.001) (4, 19). The molecular landscape among uterine serous carcinomas is an active area of investigation and characterized by a high degree of copy number alterations and frequent *TP53* mutations (80 to 90%) (20). Additionally, alterations in *FBXW7* and *CCNE1*, both involved in cell cycle regulation, have been found in 20% of uterine serous carcinomas (21, 22). *CCNE1* interacts with cyclin dependent kinase 2 (CDK2) to allow progression to G1/S cell cycle checkpoint (23). Thus these cell cycle alterations suggest potential therapeutic efficacy of CDK inhibition in *CCNE1* abnormal tumors and have been studied in a number of malignancies (24, 25). Molecular studies among endometrial tumors have identified a higher rate of *TP53* overexpression and a three-fold higher rate of *HER2* overexpression among Black patients compared to White patients (19, 26). Similar to our findings, mutations in *PTEN* (favorable prognostic indicator) were found at a significantly higher frequency among White patients compared with Black patients (27). A large genomic database study of over 2100 uterine tumors using next-generation sequencing confirmed the predominance of *TP53* mutations, and recurrent alterations of *PIK3CA*, *PPP2R1A*, *ERBB2*, *CCNE1*, *FBXW7* and *MYC* (28). However in this large dataset the correlative clinical information was not included. The role that ancestry plays in the development of aggressive tumors among Black patients is an area of investigation (29). Furthermore the impact that ancestry and chronic stress plays on the epigenome of Black patients warrants further investigation as noncoding RNAs continue to emerge as important regulators of gene expression (30). Among people of African ancestry with breast carcinoma, chronic stress related to racism, socioeconomic or environmental factors may drive stress at a molecular level (31). Under chronic stress conditions, tumor cells adapt to develop a high tolerance for stress shown through an up-regulation of adaptive stress response (ASR) genes. The Cancer Genome Atlas (TCGA) study identified 46-88 ASR genes that exhibited race-related differential expression, which represent potential therapeutic targets (31). The molecular role that chronic stress plays in uterine serous carcinoma is not clearly understood.

As we develop new cancer therapies for endometrial cancer, including uterine serous carcinoma, we must recognize potential molecular differences among races in order to develop innovative strategies (32). Over the last decade we have seen an increase in new cancer therapies available to patients with endometrial cancer (33, 34). Racial differences between clinical trial populations and “real-world” patient populations may present a challenge. Study 309-KEYNOTE-775, a phase 3 study confirmed the efficacy of pembrolizumab plus lenvatinib (Overall survival: 18.3 vs. 11.4 months; hazard ratio, 0.62; 95% CI, 0.51 to 0.75; P<0.001). However, Black patients were underrepresented (4.1% of patients in lenvatinib plus pembrolizumab arm and 3.4% of patients in the chemotherapy arm) (35). Novel therapies for uterine serous carcinoma include Adavorsertib, a WEE1 kinase inhibitor. WEE1 is a key regulatory of G2/M and S phase checkpoints, and preclinical evidence has shown promise in *CCNE1* amplified models (36). Phase II evidence of Adavorsertib reported an ORR of 29.5%; Black patients comprised 5.9% of the cohort (37). Underrepresentation of minorities in all phases of clinical trials has the potential to lead to drug approvals that are suboptimal or ineffective in the population who will receive these treatments. Therefore, it is critical that we make efforts to enroll minorities in clinical trials.

A major limitation to our study is that somatic tumor testing was only performed on advanced and recurrent endometrial tumors. A percentage of our patients were treatment naïve with advanced disease, however given our small sample size we were unable to determine if these genomic events occur at disease presentation or as a result of drug resistance. The role of racial disparities in uterine cancer outcomes is multifactorial, thus the impact *CCNE* amplification has on survival must be further investigated with these factors in mind. These numbers are small in our single institutional series, and validating these finding in a larger prospective cohort will be important. An additional limitation to our study is the use of self-identified race. Ancestry is more accurately described by large genomic databases, thus additional studies are needed to accurately describe these race-based findings (38). A strength of this study was the diverse patient population which allowed us to find meaningful racial differences between genomic alteration rates.

Uterine cancer is one of the few malignancies to show an increasing incidence over the last decade. The role that health care inequity, structural racism and discrimination plays in the rising incidence of Black patients dying from uterine cancer must not be diminished. However, through access to high quality care and research we may discover additional biological contributors to this health care gap.

In our current study we identified that Black patients are more likely to be diagnosed with high-risk endometrial subtypes with distinct molecular subtypes. Our findings highlight the need to focus on increasing minority enrollment in clinical trials and access to tumor next generation sequencing to identify actionable mutations in this high unmet need population.

## Data Availability

The raw data supporting the conclusions of this article will be made available by the authors, without undue reservation.
